# O.3.3-10 The why and how of co-production between professionals and volunteers: a qualitative study of community-based healthcare in Denmark

**DOI:** 10.1093/eurpub/ckad133.165

**Published:** 2023-09-11

**Authors:** Lene Gissel Rasmussen, Halfdan Thorsø Skjerning, Viola Burau

**Affiliations:** Research Unit for General Practice Aarhus, Denmark; Department of Public Health, Aarhus University, Aarhus, Denmark; lenegissel@ph.au.dk; Research Unit for General Practice Aarhus, Denmark; Department of Public Health, Aarhus University, Aarhus, Denmark; lenegissel@ph.au.dk

## Abstract

**Purpose:**

The purpose of the present study was to explore co-production in practice by examining how public health service and voluntary sports clubs collaborate to support people with chronic illness to participate in physical activity in a Danish community setting. Specifically, our research asked the following questions: Which institutional logics guide the practice of co-production in municipalities and voluntary sports clubs? And how do the involved stakeholders co-produce community-based healthcare? The study adopted a critical perspective on the use of co-production to promote physical activity by linking public health service and voluntary sports clubs.

**Methods:**

The study used a critical case study approach to examine the practice of co-production. The analysis was built on qualitative data from nine semi-structured interviews, two information interviews and project documents. Interviews were recorded, transcribed verbatim and coded drawing on the theory of institutional logics.

**Results:**

The findings document how the interplay between professionals and volunteers has implications in their everyday practice and offer three central insights into the practice of co-production: Firstly, in the process of co-production the interplay between different and sometimes contradictory logics may lead to conflict, dilemmas, feelings of conflicting pressures and unfulfilled expectations amongst the involved stakeholders. Secondly, the study offers examples of how different logics can be combined and balanced. Here, the stakeholders especially draw on strategies based on the network logic, which facilitates mutual understanding and matching of expectations. Thirdly, the study highlights context-specific variation, where co-production emerged as highly contingent, reflecting the dynamic interaction between logics and the context-specific management by the involved professionals and the sports club volunteers.

**Conclusions:**

This study makes an original contribution to the conceptual understanding of co-production in healthcare service, thus emphasising the benefit of paying attention to the network logic when building bridges between public healthcare service and voluntary sports clubs to promote physical activity – and to unite the seemingly contradictory “why” and “how” of co-production in practice.

**Support/Funding Source:**

No funding to declare.

Published in Journal of Sociology and Social Policy, 2022, Vol. 43 No. 1/2, pp. 197-213. https://doi.org/10.1108/IJSSP-01-2022-0027

